# Draft Genome Sequences of 33 Salmonella enterica Clinical and Wildlife Isolates from Chile

**DOI:** 10.1128/genomeA.00054-15

**Published:** 2015-03-19

**Authors:** Magaly Toro, Patricio Retamal, Marc Allard, Eric W. Brown, Peter Evans, Narjol Gonzalez-Escalona

**Affiliations:** a Center for Food Safety and Applied Nutrition, Division of Microbiology, Food and Drug Administration, College Park, Maryland, USA; b Department of Nutrition and Food Science, University of Maryland, College Park, Maryland, USA; c Facultad de Ciencias Veterinarias y Pecuarias, Universidad de Chile, Santiago, Chile

## Abstract

*Salmonella enterica* causes health problem worldwide. The relationships among strains that are from the same serotype but different hosts, countries, and continents remain elusive. Few genome sequences are available from S. enterica isolates from South America. Therefore, we sequenced the genomes of 33 strains from diverse sources isolated in Chile and determined that they were of different serotypes. These genomes will improve phylogenetic analysis of Salmonella strains from Chile and the rest of South America.

## GENOME ANNOUNCEMENT

Salmonella enterica is a major pathogen in the world, and it causes >1,000,000 cases of foodborne disease in the United States every year (1). In Chile, Salmonella is the most frequently involved foodborne pathogen in outbreaks in recent years (2). More than 2,500 Salmonella serovars are described (3), and they associate with a wide range of hosts. The pathogen causes infection not only in humans and domestic animals, but it also can infect wildlife; recent studies demonstrated the presence of Salmonella in pinnipeds, penguins, and waterfowl (4–6). Currently, only one genome sequence from Salmonella isolated in Chile is available (7). Here, we announce 33 draft genome sequences from a collection of S. enterica strains isolated in Chile from 2010 to 2012 and from different hosts and geographical locations, including 12 human clinical samples and wildlife samples.

DNA from each strain was isolated from overnight cultures with the DNeasy blood and tissue kit (Qiagen, Valencia, CA). Libraries were prepared using 1 ng of genomic DNA with the Nextera XT kit (Illumina, San Diego, CA), and the genomes were sequenced using MiSeq Illumina with the V2 kit (2 × 250 bp), according to the manufacturer’s instructions, at 40 to 190× coverage. The genomic sequence contigs for each strain were *de novo* assembled using CLC Genomics Workbench version 7.6.1 (CLC bio, Germantown, MD, USA). Ridom Seqsphere+ was used for *in silico* multilocus sequence type (MLST) analysis, and the sequences were annotated using the NCBI Prokaryotic Genomes Automatic Annotation Pipeline (PGAAP) (http://www.ncbi.nlm.nih.gov/genome/annotation_prok).

The average G+C mol% of these strains was 52.1%, similar to the reported G+C content for other Salmonella strains (8). The genome length was also within the range described for Salmonella (4.6 Mb to almost 5.1 Mb) (9). The number of contigs per assembly for each isolate ranged from 32 to 92 (Table 1). While the samples were isolated from different hosts and geographical locations, *in silico* analysis determined that they belong to only 11 sequence types (ST), most of which were already reported in the S. enterica MLST database (http://mlst.warwick.ac.uk/mlst/dbs/Senterica) (Table 1). S. enterica strain serotype Havana presented different STs (ST588 and ST1524); the remaining 9 serotypes displayed a single ST each (Table 1). Those STs agreed with the serotyping results reported for the same strains in previous studies (4, 5, 10). Moreover, we found a new ST for S. enterica serovar Paratyphi B, with a *hemD* gene differing from previously described allele 24 by one new substitution at position 270 (T instead of C). Additionally, a preliminary analysis for detecting the presence of plasmids indicated that 13 of these isolates carry plasmids (Table 1) (11). We used two approaches, PlasmidFinder and pMLST (https://cge.cbs.dtu.dk/services); the first detects plasmid replication origins, and the second determines incompatibility types, both allowing mining for contigs with those characteristics.

**TABLE 1:** Metadata for S. enterica subsp. *enterica* strains isolated in Chile from different hosts

CFSAN no.	Isolate name	WGS accession no.^*a*^	Source	Serotype	No. of contigs	ST	PlasmidFinder/pMLST^*b*^
CFSAN024756	SAG1	JWQW00000000	Penguin	Agona	55	13	−/−
CFSAN024757	SAG2	JWQV00000000	Kelp gull	Agona	55	13	−/−
CFSAN024758	SAG3	JWQU00000000	Penguin	Agona	64	13	−/−
CFSAN024759	SAG4	JWQT00000000	Clinical	Agona	60	13	−/−
CFSAN024760	SAG5	JWQS00000000	Clinical	Agona	48	13	−/−
CFSAN024761	SAG6	JWQR00000000	Penguin	Agona	52	13	−/−
CFSAN024763	SAN3	JWQP00000000	Clinical	Anatum	32	64	−/−
CFSAN024764	SAN4	JWQO00000000	Clinical	Anatum	60	64	−/−
CFSAN024765	SBR1	JWQN00000000	Sea lion	Brandenburg	38	65	−/−
CFSAN024767	SDU1	JWQM00000000	Kelp gull	Dublin	47	10	+/+
CFSAN024768	SDU2	JWQL00000000	Clinical	Dublin	37	10	+/+
CFSAN024769	SDU3	JWQK00000000	Clinical	Dublin	35	10	+/+
CFSAN024770	SHA1	JWQJ00000000	Sea lion	Havana	49	1524	+/+
CFSAN024771	SHA2	JWQI00000000	Gray gull	Havana	41	588	+/−
CFSAN024773	SHE2	JWQG00000000	Kelp gull	Heidelberg	35	15	+/−
CFSAN024774	SHE3	JWQF00000000	Kelp gull	Heidelberg	44	15	+/+
CFSAN024776	SHE5	JWQE00000000	Clinical	Heidelberg	38	15	+/−
CFSAN024777	SHE6	JWQD00000000	Clinical	Heidelberg	39	15	+/−
CFSAN024778	SIN1	JWQC00000000	Kelp gull	Infantis	41	32	−/−
CFSAN024779	SIN2	JWQB00000000	Kelp gull	Infantis	47	32	−/−
CFSAN024780	SIN3	JWQA00000000	Kelp gull	Infantis	42	32	−/−
CFSAN024781	SIN6	JWPZ00000000	Clinical	Infantis	44	32	−/−
CFSAN024715	SIN7	JWRH00000000	Clinical	Infantis	39	32	−/−
CFSAN024716	SLI1	JWRG00000000	Sea lion	Livingstone	32	457	−/−
CFSAN024717	SLI2	JWRF00000000	Sea lion	Livingstone	41	457	−/−
CFSAN024718	SSE1	JWRE00000000	Kelp gull	Senftenberg	32	14	−/−
CFSAN024719	SSE2	JWRD00000000	Kelp gull	Senftenberg	43	14	−/−
CFSAN024720	SSE3	JWRC00000000	Kelp gull	Senftenberg	56	14	−/−
CFSAN024721	SSE4	JWRB00000000	Clinical	Senftenberg	52	14	+/+
CFSAN024722	SSE5	JWRA00000000	Clinical	Senftenberg	45	14	+/−
CFSAN024723	SSE6	JWQZ00000000	Kelp gull	Senftenberg	72	14	−/−
CFSAN024724	SSE7	JWQY00000000	Kelp gull	Senftenberg	92	14	−/−
CFSAN024725	SGB1	JWQX00000000	Kelp gull	Paratyphi B	58	New	−/−

^a^ WGS, whole-genome shotgun.

^b^ PlasmidFinder/pMLST, presence of plasmids. +, positive; −, negative.

The data provided will help in understanding the differences between Salmonella strains isolated from different countries and continents, improving traceback investigations for foodborne-related outbreak events. Moreover, these new draft genome sequences will contribute to the analysis of host-associated differences among Salmonella strains and provide phylogenetic insights into their evolution on different continents. A detailed report of these genomic features will be addressed in a future publication.

### Nucleotide sequence accession numbers.

The draft genome sequences for these 33 Salmonella isolates are available in GenBank and are listed in Table 1.
